# A qualitative study of nurse‐patient communication and information provision during surgical pre‐admission clinics

**DOI:** 10.1111/hex.13270

**Published:** 2021-06-05

**Authors:** Dominic Roche, Aled Jones

**Affiliations:** ^1^ School of Healthcare Sciences Cardiff University Cardiff UK

**Keywords:** ambulatory care, enhanced recovery after surgery, general surgery, nursing, outpatients, patient activation, patient engagement, patient information, patient involvement, patient participation, patient safety

## Abstract

**Background:**

Health‐care service users are often being described as ‘co‐producers’ with an active role in their care. However, there are challenges associated with this approach, including how standardization affects personalized care, and the ability of patients to retain high volumes of information.

**Objective:**

Our study explores patient and nursing perspectives of information provision in the pre‐admission element of an Enhanced Recovery After Surgery programme, an evidence‐based approach implemented to improve the quality of surgical care. Our analysis has been informed by an evidence‐based model developed by Grande et al Patient Educ Couns. 2014;95:281.

**Design/Setting and participants:**

This was a qualitative study including observations of pre‐admission clinics and semi‐structured interviews across three surgical wards. Patients (n = 21) and registered nurses (n = 21) were purposively selected for interviews.

**Results:**

Patients welcomed the opportunity for active involvement in their care. However, we also identified informational boundaries and how illness and treatment‐related anxieties were barriers to patient engagement with the information provided.

**Discussion:**

We recommend that to support a patient‐centred and individualized approach to patient involvement the ‘information (giving) + activation’ element of Grande et al Patient Educ Couns. 2014;95:281 model be reconfigured to allow for ‘information (giving) + *exploration* +activation’.

**Conclusion:**

Nurses need to feel empowered to adopt strategies that allow for different informational needs, rather than adopting a one‐size‐fits‐all paternalistic approach.

**Patient contribution:**

This study focused on patient involvement and we give thanks to all the patients who took part in interviews and those who allowed us to observe their care.

## 1 INTRODUCTION

Historically, relationships between patients and health‐care professionals have followed a paternalistic model in which patients are regarded as passive recipients of care.^1^ Anderson and Funnel^2^ describe an ‘acute care paradigm’ which has underpinned the approach to health care in hospitals, where patients surrender control to health‐care professionals who are then relied on to use their expert skills and knowledge to solve patients’ health problems. This paradigm closely reflects the notion of the ‘sick role’,^3^ which positions an acutely ill person as temporarily passive, while being treated by an active doctor and other carers. However, more recent thinking has questioned the sick role as a useful explanatory concept, suggesting that it is more appropriate to view health‐care service users as co‐producers with an active role in their care.^4^, ^5^

Within health‐care academia, practice and policy, this shift in focus has led to the development and use of strategies and interventions to actively involve patients in supporting safe practice and reducing harm. ^6^, ^7^, ^8^, ^9^, ^10^ A recent example is the Enhanced Recovery after Surgery (ERAS) programme, an evidence‐based ‘bundle’ approach implemented internationally to improve the quality and safety of patient care for major surgery.^11^ A prevalent aspect within ERAS is the active involvement of patients in the delivery of their care,^12^ for example ensuring patient involvement during early and continuing postoperative mobilization, which can reduce the risk of postoperative complications including pulmonary and thromboembolic complications.^11^, ^13^, ^14^ This is different to ‘traditional’ surgical care, as postoperative mobilization occurs much earlier and prioritizes direct patient involvement in this aspect of rehabilitation.

**FIGURE 1:**
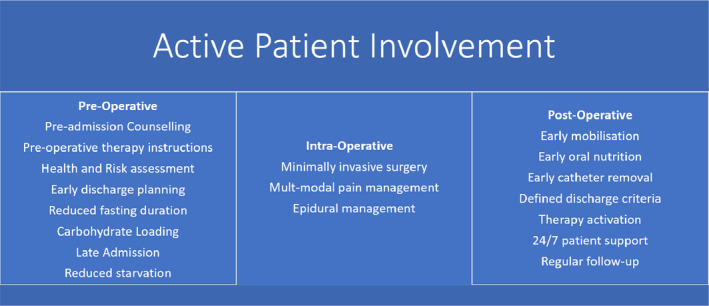
An overview of patient involvement in an ERAS programme

An overview of patient involvement in an ERAS programme is provided in Figure 1.

An important factor affecting the willingness and ability of patients to become involved in safety initiatives is that patients need to be aware of any requirements for involvement and what this will entail in terms of specific actions and behaviours that may be required by patients.^9^ In line with this, patients who are familiar with their own care and treatment plans are more likely to become involved in safety‐related initiatives.^15^ Previous studies have also reported the importance of health professionals managing patients’ expectations by clearly communicating what is expected of them during their care. For example, the provision of good quality information to patients can provide patients with a sense of control^16^, ^17^ and this can act as a key facilitator in making the shift from passive to active patient in the context of postoperative recovery.^18^

The overall aim of this paper was to present findings from the analysis of qualitative interview and observational data to explore patient and nursing perspectives of information provision in an ERAS programme. Our analysis is informed by Grande et al^19^ model, which posits that patient involvement initiatives are more likely to be achieved when they fit within existing workflows, require little additional human resources and proportional amounts of work by patients. Specifically, the model proposes a ‘Information +Activation’ approach where health professionals target patient involvement through encouragement, prompting, coaching, help and support to perform specific actions in the clinical encounter. In this sense, the facilitator role adopted by the health‐care professional is critical during the encounter in finding a ‘sweet spot’, which generates improved knowledge in patients and, in turn, motivates active involvement.^19^

However, potential tensions exist in that the ‘help and support’ offered by professionals is bounded and determined by the performance of specific behaviours expected of patients enrolled in ERAS. For example, early postoperative mobilization largely occurs as a pre‐determined requirement of the ERAS specification, rather than unconditionally resulting from the exploration and planning of patient identified goals. Although clinical outcomes of the ERAS programme have been published internationally,^20^ there is little research exploring the so‐called ‘sweet spot’ and the ‘information +activation’ approach in practice. One exception is a recent qualitative study of health‐care professionals’ views of ERAS,^18^ which identified a number of implementation challenges, including how standardization affected personalized patient care, and challenges associated with the high volume of information provision impacting on patients’ ability to retain information.

The specific aims of the paper are to address gaps in existing understanding through exploration and critique of.

- the ‘information +activation’ model in the context of the pre‐assessment element of an ERAS programme, by analysing nurses’ attempts (and patients’ reactions) to manage patient expectations and prepare patients for an ‘active’ postoperative role.
- the tensions between standardized programmes of care delivery, such as ERAS, and individualized patient needs and whether these tensions impact on the receptiveness of patients to the information +activation approach.

In doing so, we contribute to the better understanding of some of the theoretical constructs that abound in patient involvement, while also giving a better understanding of the ‘inner workings’ of the internationally implemented ERAS programme from a patient and nurse perspective.

## 2 METHODS

Ethical approval for the study was granted by the South East Wales Research Ethics Committee (reference: 12/WA/0192). Approximately 18 hours of observations were recorded across 11 separate outpatient clinics and 42 interviews took place across three hospital wards in Wales, specializing, respectively, in upper gastrointestinal, colorectal and orthopaedic surgery. Table 1 provides summary detail of interview participants. Observations, then interviews were undertaken by the first author across the three specialties, with interview participants split equally between patients and nurses. Potential participants were provided with written information about the study prior to agreeing to take part. All participants met the inclusion criteria that they were involved in an ERAS programme, were aged over 18 and able to provide informed consent. Assurances were provided to all participants about protection of anonymity and confidentiality. Interviews with nurses took place in offices and other private spaces on the respective wards. Patient interviews took place at their homes.

**TABLE 1:** A summary of participants

	Nurse Agenda for Change (AfC) pay band*	Nurse gender	Patient age	Patient gender
Upper gastrointestinal	3 × AfC Band 5 2 × AfC Band 6 2 × AfC Band 7	1 Male 6 × Female	2 × 45‐50 Years 1 × 55‐60 Years 1 × 55‐60 Years 2 × 65‐70 Years	6 × Male
Colorectal	1 × AfC Band 3 2 × AfC Band 5 3 × AfC Band 6 1 × Band 7	2 × Male 5 × Female	1 × 50‐55 Years 2 × 55‐60 Years 3 × 60‐65 Years 2 × 65‐70 Years	2 × Male 6 × Female
Orthopaedic	4 × AfC Band 5 3 × AfC Band 6	1 × Male 6 × Female	1 × 45‐50 Years 2 × 50‐55 Years 2 × 55‐60 Years 2 × 65‐70 Years	3 × Male 4 × Female

* *Agenda for Change* is the national pay system for all NHS staff, with the exception of doctors, dentists and most senior managers.

Semi‐structured interviews were chosen as they are a widely used qualitative method that enabled the researchers to identify topics of interest, whilst also allowing for discussions directed by participants responses.^21^ Structured observations were purposively sampled to include ERAS patients and data from these clinics were recorded by the first author based on a list of prompts developed by the researchers, which allowed interactions in pre‐assessment appointments to be captured in a systematic way.^22^ The use of multiple data collection methods can help add rigour, breadth and depth to a study.^23^, ^24^

Triangulation was a feature of data analysis, which involved the comparison of data relating to the same phenomenon but derived from different phases of fieldwork and the accounts of different participants. Triangulation is generally considered a process of using multiple perceptions to clarify meaning, allowing the researcher to verify the repeatability of an observation or interpretation.^21^, ^25^ Initial thematic analysis of the data was undertaken by the first author, guided by a six‐step process^26^ which entailed familiarization with the data and generating initial codes, which were subsequently used to produce a thematic ‘map’. On‐going analysis and discussion between the two authors resulted in further iterations and refinement of the map which proved useful in comparing our emergent findings to the extant literature, including Grande's model.^19^

### 2.1 Findings

The findings are presented in two sections. The first, ‘exploring and critiquing information +activation’, examines the pre‐admission interactions that took place between nurses and patients. All wards in the study adhered to ERAS guidelines recommending the use of pre‐operative patient information as a mechanism to encourage patients to become active participants in their postoperative recovery.^11^, ^13^, ^14^ Here, we focus on hitherto unexplored strategies enacted by nursing staff to attempt to manage patients’ expectations and to prepare patients to undertake this active role. The next findings section, ‘informational boundaries’, considers the tensions between the attempts of nurses to provide standardized information or elements of ERAS, alongside the specific needs and wants of some individual patients. We focus on how these perceived boundaries can influence patient receptiveness to the information and in turn impact on attempts to ‘activate’ patients. The data included in this paper are selected to be illustrative of the sample population in relation to the themes and discussion presented. This is guided by Braun and Clarke's framework,^26^ where we have striven to provide vivid examples of the points demonstrated. Data extracts are labelled according to whether the interviewee was a patient or registered nurse (RN), and the relevant clinical specialty.

### 2.2 Exploring ‘information +activation’

In this first section, we show that the ‘information +activation’ process was, in many cases, an effective approach to raising patient awareness and managing patient expectations of their anticipated role within the ERAS programme. For example, prior to admission for surgery, patients meet with members of the multidisciplinary team, including a RN, who provide the information necessary to support and guide patients through the planned surgical process. RNs described how during this appointment they sought to manage patient expectations to help prepare them for an ‘active’ approach to postoperative care, in line with the ‘information +activation’ model.^19^ Patients also described how information provided during the pre‐admission appointment was helpful in managing expectations and preparing them for the upcoming hospital care.

It’s kind of getting rid of those preconceptions […] pre‐assessment kind of busts those myths […] They’re not surprised when we get them moving quicker. They’re not surprised when they’re going home a lot quicker. [Colorectal: RN4]

Well yeah, that’s part of ERAS, cos they tell you exactly what’s going to happen […] It took away completely the fear of the unknown. I should imagine a lot of patients go in and they don’t know what happened or why. It took away, I knew exactly what was gonna happen. [Upper GI: Patient 5]

Early postoperative mobilization and reduced length of hospital stay greatly reduce the risk of iatrogenic harm occurring to patients.^11^, ^20^ During pre‐admission, patients were provided with information about how adopting an active role in their postoperative care could help manage these risks. In addition to providing information about early mobilization, ERAS guidelines also highlight the importance of providing pre‐admission information to patients about discharge goals and predicted length of hospital stay. ^11^, ^13^, ^14^ Patients and nurses described how this information helped to manage expectations during subsequent hospital care, demonstrating a shared awareness and expectations of postoperative care.

Our aim is to get you in and out as quickly as possible. But safely. […] Our message is the less time you are in hospital the better it is for you. [Orthopaedic: RN7]

It’s about patient expectation changing as well. You know, you do learn all about the enhanced recovery programme and what we do and all the benefits of it. And the benefit of it is to get you home earlier. That’s our ultimate goal. [Colorectal: RN6]

Well, I certainly understood the whole basis of the programme […] why it was important and the success they had from it. Yes, that was all explained to me […] which is why I think I was so keen to do it […] (less chance) to pick up infections, etcetera. [Colorectal: Patient 7]

It was all to do with infections and blood clots and the rest of it. Oh yes, that was all explained to me very clearly […] I knew exactly what they were doing and why it was important [Orthopaedic: Patient 7]

The ‘information +activation’ approach adopted by RNs during pre‐assessment helped to raise patients’ awareness of the expectations for an active role in postoperative care. Patients valued the information provided and were positively receptive to the prospect of early mobilization. Patients also mostly welcomed the enhanced awareness of potential risks and the advantages associated with shortened length of hospital stay. From this, it is possible to conclude that the ‘information +activation’ model was effective in terms of raising patient awareness and managing expectations of the ERAS programme.

However, deeper analysis of these initial surface‐level conclusions demonstrates that, when analysed from an individual patient perspective, interactions during these pre‐admission appointments can be more complicated. This is explored in the next theme in which we discuss ‘informational boundaries’ and how these can impact on and influence the nurse‐patient interactions, adding more depth to these initial conclusions.

### 2.3 Informational boundaries: Procedural and professional domains

Despite many patients describing the usefulness of the information provided, it was evident during pre‐assessment observations that some patients were, understandably, distracted by their diagnosis and possible prognosis and required information beyond the defined scope of the ERAS protocol. For example, one patient was concerned about the results of chemotherapy and radiotherapy in the lead‐up to the planned operation and was keen to find out about a scan that had taken place. Despite the patient being clearly pre‐occupied with these questions and uncertainties, the nurse coordinating the appointment was unable, or unprepared to answer these questions [Field Notes: Colorectal Pre‐assessment].

Other patients wanted to find out details about their upcoming operation; for example, in one pre‐assessment appointment, a patient wanted to find out whether they were likely to have a stoma as a result of their operation. The nurse did not, or was unable to, provide details or assurances about this [Field Notes: Colorectal Pre‐assessment]. This was quite common in the pre‐assessment appointments observed when patients asked questions about their procedure and nurses were unable or unwilling to provide specific details. In another example, a patient asked about wound healing and was given the rather broad response ‘everyone heals differently’ [Field Notes: Orthopaedic Pre‐assessment]. At times RNs made it clear, these types of uncertainties would remain until the operation itself ‘It's all ifs and buts till you get down there’ [Field Notes: Colorectal Pre‐assessment]. It was also acknowledged by one nurse that patients would want this type of information ‘they'll want to know about seeing the anaesthetist and seeing the doctors and what needs be done’ [Upper GI: RN3]. So, although these appointments were framed within the wider health‐care journey that patients were experiencing, it was clear there were ‘informational boundaries’ in place relating to the information that some patients required, compared to the information that nurses were willing or able to share.

Our analysis of field observations demonstrates that patients attended the pre‐assessment appointment with information needs that were not directly, or satisfactorily addressed by nurses. However, unbeknown to patients the appointment was not the designated to discuss specific details about surgical procedures, a point summed up in the following quote.

We don’t really go through the procedure they’re having […] the medic normally goes through the procedure […] That’s done normally at the bedside the day before they come in. So, they get more information when they’re consented, about their procedures [Colorectal: RN3]

To summarize, the data show two boundaries or overlapping ‘no‐go’ zones during the information provision, one about procedure and the other about prognosis. The common ground to both being uncertainty; that is, the nurse either does not know, or cannot share the information. The data also show there to be professional boundaries between nursing staff and medical staff – with each providing different information at different times during the patient journey, as shown when the nurse invoked some information as being in the medical domain to be dealt with during the consent process. Equally revealing is that patients’ attempts to breach these boundaries demonstrate their lack of awareness or understanding about these boundaries and the purpose of the pre‐assessment appointment – that it is about basic information provision, not treatment discussion or exploration.

### 2.4 Informational boundaries: Patient anxiety

Patient anxiety and information overload were also identified as boundaries to the effective exchange of information. In some cases, patients were simply not receptive to the provision of information, irrespective of the nature of this information. This is demonstrated in the following example of observational data, in which a patient remarked there was ‘too much information’ and told the nurse ‘I just want to come in and have it (the operation) done’ [Field Notes: Colorectal Pre‐assessment]. During this particular appointment, the patient commented two further times that too much information was being provided. Similar examples were observed at other pre‐assessment appointments, where one patient remarked to the nurse delivering the information ‘I don't want to go too deep or I’ll start worrying’, whereas another commented that she was happy there was not much time to think about the operation ‘don't start thinking about it, just get it over with’. [Field Notes: Upper GI Pre Assessment]. However, on these and other occasions, this did not deter nurses who continued to deliver the ‘required’ information throughout the appointment. Nevertheless, during interviews nurses reflected that patients were potentially overwhelmed by the amount of information delivered, with one commenting that patients ‘sometimes […] get too much information’, whilst at the conclusion of a pre‐assessment clinic, another nurse announced to the patient that ‘I’ve bombarded you a bit’. [Field Notes: Colorectal Pre‐assessment].

In addition to the volume of information, patients’ fear and anxieties were also a barrier to successfully receiving pre‐admission information.

I was traumatised that I was going to have it done […] that’s the hard part, really. The sort of accepting that that’s what’s happening […] I was so scared I think my brain stopped working. It really was a bit of a blur […] And you don’t know how bad it is. Although they assured me as much as they could […] It’s a job to face the fact that you’ve got cancer […] I find too much knowledge is not a good thing. [Colorectal: Patient 8]

Yeah. I can’t remember. […] Over my head, I don’t know. It was like I was so anxious as well; I didn’t take it all in. [Upper GI: Patient 1]

However, nurses were aware patients might be distracted, anxious or fearful about their diagnosis and upcoming surgery and that this could prove a barrier to the delivery and receiving of information.

They’re nervous about coming in, you know. Anxious about the operation and they’ve got a lot of things on their mind anyway. [Upper GI: RN1]

And they’ve had a devastating diagnosis as well. And prognosis. So, they’ll be dealing with all that fear […] As well as being a huge operation which is – they’re only having it because it’s potentially curative. [Upper GI: RN3]

Despite this awareness, nurses did not deviate from pre‐admission clinic ‘script’ when any of the field observations were undertaken.

I think they get so much information. […] If you’re coming to terms with a diagnosis of cancer, for instance, you’ve only just come to terms with that. Or have you come to terms with that diagnosis? […] I think sometimes it can be a little bit too much to take it all in. When you’ve come and you’re nervous […] the majority of people would be nervous going to a hospital appointment, so how much (do) they retain? [Colorectal: RN4]

However, striking a balance between too much, too little or the right amount of information did not appear easy. There were differing views from nurses about how these potential barriers to information receptiveness should be addressed. One acknowledged that there was a risk of ‘pushing’ information onto patients, but also stated that it was important that patients understood ‘certain things’.

Not everybody wants to know everything about […] what’s going to happen. And we take that into account as well and I hope we wouldn’t […] push people into knowing things they wouldn’t want to know […] But there’s still certain things that it’s important they understand [Colorectal: RN6]

This introduces another informational boundary issue, or tension, between providing too much information and providing the appropriate or ‘right’ information. Overall, there was evidence that although patients welcomed information and the opportunity to take a more active role in their care, some were clearly distracted and burdened by their illness and specific information needs, which meant that some information about postoperative care was a low priority for them. An important issue identified from our findings is the concept of ‘information boundaries’ and the rendering of certain ‘scripted’ responses ineffective, which are further explored in our discussion section.

## 3 DISCUSSION

Our findings show that patients were provided with information during a pre‐admission appointment and that this information encouraged patients to prepare for an active role during their surgical recovery. In most cases, patients welcomed this information and the opportunity for active involvement in their hospital care. However, we also identified a variety of informational boundaries which shaped pre‐admission appointments. Boundaries were identified by nurses which pre‐determined which professional groups shared information with patients, and at the specific points in the patients’ journey that this would occur. Boundaries were also identified by patients which demarcated their preferences for the amount and/or timing of information that should be delivered.

In most cases, a standardized ‘scripted’ information‐giving approach was deployed by RNs during the pre‐assessment appointment and nurses were unable, or unwilling, to deviate from this approach, even when patients requested specific information and despite nurses’ apparent awareness of the perils of ‘too much information’ during the clinical appointment. Knowing and understanding the individual concerns and needs of patients are clearly important as they directly relate to how effectively patients are able to absorb and process new information. In this sense, the requirement to deliver standardized information in a way that also addressed individualized information needs is a key message from these findings. This implies that a step is required between ‘information’ and ‘activation’ in the model posited by Grande et al^19^

Here, we further discuss our contribution to understanding the inner workings of this model and some of the complexities involved when applying the model to a programme such as ERAS. The following section explains that, based on our findings, to more effectively support a patient‐centred and individualized approach to patient involvement the ‘information (giving) + activation’ element of Grande et al's model^19^ could be reconfigured to allow for ‘information (giving) + *exploration* +activation’.

### 3.1 Exploration as a possible way forward?

In Grande's model,^19^ the onus is on the provider to effectively facilitate interaction. However, the current study findings indicate that facilitation was not a role that nurses in our study were willing (or able) to adopt. So, although attempts to encourage patients to take an active role in postoperative care are a good fit in terms of the existing workflow, with no additional human resources required, this resulted in the interactions being rendered ‘passive information provision’ on the scale developed by Grande.^19^ Such unidirectional transmission of information provision characterizes patients as passive receivers of information. To support and encourage nurses to adopt a more flexible and exploratory role in their consultations with patients, we will draw on relevant literature to support our proposed addition of an intermediate ‘exploration’ phase to the Grande model, whilst also generating broader reflections on patient involvement in health care of relevance to other professional groups and circumstances.

**FIGURE 2:**
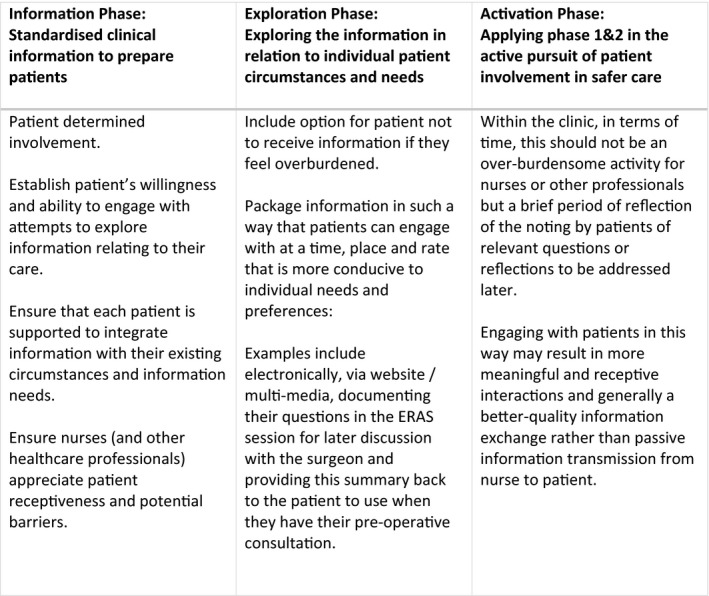
A proposed model of information +exploration = activation

By ‘exploration’, we mean a phase extending out of standardized information giving which considers individualized patient contexts and related informational needs. Figure 2 outlines how each phase is built upon towards activation of patient involvement within the context of ERAS. These phases are underpinned by Grande's^19^ recommendation that health professionals encourage, prompt and coach to help ‘activate’ patient involvement. The proposed introduction of an intermediate ‘exploration phase’ can lead to a co‐produced activation phase which is informed and sensitive to individual patient circumstances and needs, rather than the scripted and standardized activation phase seen in our findings.

Murray et al^27^ identified that it is likely patients are at least passively information receptive at some point, and involvement mediated by health‐care professionals can influence the next step of patient‐determined involvement. This of course is dependent on whether the patient is able and willing to engage with these attempts to mediate and, as our findings show, there were instances where this was not the case. Therefore, the exploration phase is important as it ensures that each individual patient is facilitated to integrate the information with their existing circumstances and informational needs. This also ensures that nurses and other health‐care professionals are in tune with patient receptiveness and potential barriers to this. The exploration phase should also include the option of patients choosing not to receive information if they feel overburdened or opting for the information to be delivered in a different way, for example electronically, via a website or documenting their questions in the session for later discussion with the surgeon and providing this summary back to the patient to use when they have their pre‐operative consultation. As a result, exploration results in information being received by patients at a time, place and rate that is more conducive to individual needs and preferences.

In terms of time, the exploration phase should not be an over‐burdensome activity for health‐care professionals, but a brief period of reflection of the noting by patients of relevant questions or reflections to be addressed later. Engaging with patients in this way may result in more meaningful and receptive interactions and generally a better‐quality information exchange, with patients and nurses working together to identify necessary information, rather than passive information transmission from nurse to patient.^17^ This approach is further reinforced by a study^28^ which found that successful attempts to involve patients in their safety relied on the quality of the patient‐clinician relationship. In addition, Sutton and colleagues^29^ present evidence which shows health‐care staff are supportive of approaches that encourage co‐operation, with other studies reporting that the encouragement, approval and positive attitudes of health‐care staff are crucial in preparing patients for an active role.^15^, ^30^, ^31^ Further to this, studies report that to encourage patient involvement information should not be based on standardized procedures, but should consider patients’ skills, knowledge, ability and specific needs, combined with appropriate explanations.^32^, ^33^, ^34^

However, paradoxically, in the quest for an ‘active’ patient, it was sometimes the case in our findings that delivering information as a script was akin to a paternalistic approach to health‐care care – ‘the patient needs to know this, even if they're not receptive’ – the antithesis of patient involvement. What is clear and of importance from our findings is that some patients were not aware or did not understand the unidirectional flow of information in these interactions. As a result, patients tried to engage in a more exploratory style of interaction, only to belatedly discover, for example, that this was not the place to explore treatment options or results. Interestingly, nurses knew that some patients desired more interaction, but were unwilling to change their approach. The new ‘exploratory phase’ we propose can help nurses facilitate more active patient involvement and exploration of information provided. Encouraging patients to think about and relate the information to their own personal circumstances and needs is more likely to create rapport and increase the quality of the interaction.

There are clearly tensions between the provision of standardized care through a programme such as ERAS and the provision of individualized care and this is not an issue unique to our study. Ultimately, any activation of information provided is dependent on whether patients are receptive to this information and what their specific informational needs might be at the time. Herbert et al^18^ explored health‐care professionals’ views of ERAS and one of the challenges identified by staff was that although the evidence base for the programme was seen as legitimate, with obvious benefits to the quality and safety of patient care, standardization affected their ability to provide personalized patient information. Our study presents similar findings to that of Herbert et al^18^ with staff reporting concerns about the volume of information they were expected to present to patients, alongside an expectation that nurses would adhere to the ERAS protocol, coupled with the struggle of wanting to provide individualized care. It is unsurprising to find that patients informational needs differ^35^; however, what is interesting is nurses’ acknowledgement that patients have these differing needs, but an apparent reluctance to act on this. This provides further evidence for the exploration stage proposed and perhaps promoting this additional step in the model will encourage nurses, and just as importantly, managers and leaders of health‐care organizations, to act on these insights.

The ERAS programme is designed to encourage patients to take a more active role throughout their health‐care journey and the information provided to patients about their risks and requirement to ‘actively’ rehabilitate after surgery could be viewed as an empowerment approach. In this sense, the inter‐personal dimension of empowerment described by Aujoulat et al (2007) is of relevance to our findings, as there were clearly attempts from health‐care providers to communicate with and inform patients in an attempt to share, to varying degrees, knowledge, values and power. Attempts to empower patients through this type of strategy have been shown to result in patients adopting a more active role in their care (for example, Chang et al 2012). As Aujoulat et al (2007) state, this approach views empowerment as an interactive process intended to develop and reinforce certain abilities in people in relation to their care, for example the ability to determine personal goals and define strategies to achieve these goals, to develop and encourage patient motivation, to seek information and to ask questions (Aujoulat et al 2007). In support of this, studies have shown that patients welcome the opportunity to ask personalized questions.^17^ However, examples of this appear to be rare in the literature, for example in the systematic review reported by Sibbern et al^36^ the authors identified that some patients felt they were not given sufficient time during pre‐admission appointments to ask relevant questions, while other patients reported that they would have liked more in‐depth verbal explanations during their pre‐admission appointment. This further supports the necessity for an exploration phase and raises questions about how much patients who desire exploration currently take from the appointments as their expectations are being only partly met, or indeed not met at all. Our findings show that nurses could have an important role to play in ensuring an effective balance between these seemingly competing priorities. The task is that of understanding when standardization is appropriate and when it is not and then being able and willing to act on this. However, in our findings, despite nursing staff identifying patient issues and acknowledging the limitations of the information‐giving approach, most failed to react to this.

### 3.2 Implications for research and clinical practice

To support our proposed model and associated approach to the provision of patient information, more clarity is needed about what is ‘allowed’ or expected in pre‐operative appointments. Specifically, more clarity for the patient that the appointment is information giving and may involve lots of information delivery, rather than information exploration or exchange. However, the latter needs to be facilitated elsewhere in a different forum or in a different way. In addition, patients who feel overburdened with information can be provided with information to take home, or web‐based information and links to consultations at a later stage, when they feel better disposed to take this information in. What is crucial is that nurses who engage with patients during these appointments are able to assess and react to the individual requirements of each patient and tailor their approach to information sharing in accordance with these requirements. This is no easy feat, as we have discussed, due to the volume of information and the drive for efficiency, particular in terms of human resources and time.

This paper is specifically focused on the interactional dynamics and accomplishment of the pre‐assessment appointment rather than reflecting on educational delivery and accordingly, we have positioned our paper in this context. However, we acknowledge there is potential to examine ERAS through the lens of educational theory, which warrants further exploration in future research. Further research could also explore what it is about individual nurses that inhibit them from providing individualized care, and conversely, what is it about those nurses who feel empowered to tailor their consultations with patients. Are there particular individual or organizational factors that can influence this and how can these be overcome (or harnessed)? In support of this, further research could see researchers taking the three‐step approach we have proposed into clinics and other areas of care to test this model in action to see if it a) prompts nurses and patients to reflect and b) whether pausing the fairly relentless provision of information to take time to reflect makes things challenging in terms of time management, or if any adverse unintended consequences are introduced which are difficult to predict outside of a ‘real world’ environment.

## 4 CONCLUSION

Nurses must consistently be alert to the fact that humans are sensitive information processors and when bombarded with lots of potential or actual distractors, the information provision leading to information retention and subsequent activation is too linear and causal an approach to base the success of informational interventions on. What we have seen in our findings is that this approach does work most of the time, for most patients, but nurses need to feel empowered to adopt strategies that can allow for different informational needs and communication challenges, rather than a one‐size‐fits‐all paternalistic approach. This further supports our proposal to adapt the model to include the opportunity for an exploration of patients’ information needs.

## CONFLICT OF INTEREST

Both authors declare that there is no conflict of interest either in this study or in this paper.

## Data Availability

Data available on request due to privacy/ethical restrictions: The data that support the findings of this study are available on request from the corresponding author. The data are not publicly available due to privacy or ethical restrictions.
